# Environmental Considerations in Cardiovascular Risk Assessment and Prevention

**DOI:** 10.1016/j.jacadv.2023.100361

**Published:** 2023-05-26

**Authors:** Sadeer Al-Kindi, Candice K. Silversides

**Figure:**
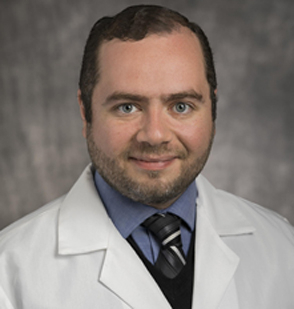

**Figure:**
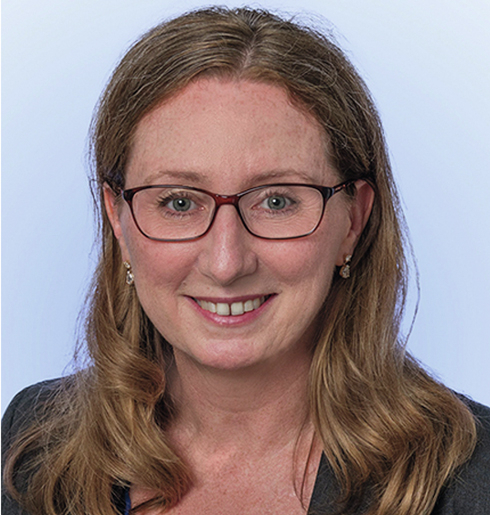

Complex interactions between genetic predisposition and the environment contribute to the development and progression of cardiovascular disease. Prevention of atherosclerotic cardiovascular disease complications, eg, has been based on the identification and treatment of traditional risk factors such as smoking or physical activity. Comprehensive preventive strategies should, however, address environmental risk factors, such as air pollution, that affect cardiovascular health.

Air pollution resulting from human activity, such as fossil fuel burning in motor vehicles, household devices, and industrial facilities, produces a large amount of air pollutants particulate matter (the most detrimental being fine particles [PM_2.5_]) and other gaseous pollutants. Inhaled particulate matter can trigger an oxidative stress response resulting in endothelial dysfunction, inflammation, autonomic imbalances, prothrombotic effects, central nervous system effects on metabolism and hypothalamic-pituitary-adrenal axis activation, and epigenomic changes.^1^ All of these factors can contribute to both short- and long-term adverse cardiovascular events including cardiovascular mortality, myocardial infarction, heart failure, arrhythmias, and stroke. Chronic exposure also increases the risk of development of traditional risk factors for cardiovascular disease, including hypertension and diabetes.^1^ Patients with an increased atherosclerotic burden are thought to be particularly sensitive to the effect of air pollution because of its effect on atherosclerosis progression and plaque vulnerability.

In this issue of *JACC: Advances*, Motairek et al^2^ studied more than 73,000 U.S. veterans who had undergone percutaneous coronary interventions and showed an increased risk of major adverse cardiovascular events and life years lost with higher long-term exposure to fine particulate matter pollution. Patients who lived in areas with relatively higher exposure (PM_2.5_ 10 μg/m^3^) had a 1- to 2-year shorter estimated lifespan when compared with those living in areas of low exposure (PM_2.5_ of 5 μg/m^3^). Air pollution had a more pronounced effect in patients who underwent percutaneous coronary interventions at younger ages. Studies such as this help to highlight the importance of air pollution on cardiovascular outcomes, especially in vulnerable populations.

Although cardiologists are familiar with discussing and treating traditional cardiovascular risks factors, environmental factors, such as air pollution, are often overlooked. For high-risk patients, especially those with pre-existing cardiovascular disease who are exposed to high levels of pollution, patient education and treatment strategies should be discussed.^3^ Patients should be aware of strategies to reduce in-home penetration of polluted outdoor air with indoor air purifiers and closed windows. Air purifiers can lower fine particulate matter by more than 50%. Wearing masks when outdoors or avoiding commuting during rush hour may be other helpful approaches for patients living in areas with significant air pollution. Although studies are limited, dietary modifications and exercise may mitigate the effects of air pollution.^4^^,^^5^ Other strategies, such as air pollution sensors, may help personalize the approach to risk assessment and treatment in the future. Although an individual approach is important, larger societal and governmental approaches are needed to improve air quality as well.^1^

Health care provision itself is a major source of air pollution and greenhouse gas emission, contributing to climate crisis and human health. Policies to reduce air pollution can also affect climate change. Even some of the changes in health care policies can have positive impacts on air pollution and the climate. In this same issue of *JACC: Advances*, Bawa et al^6^ show a reduction of 12,518 metric tons of greenhouse gas from gasoline when remote cardiac monitoring of patients with cardiac implantable electronic devices was used instead of conventional monitoring, equivalent to approximately 15,000 acres of U.S. forests saved, and 208,000 trees planted for 10 years in addition to significant cost savings.

Although we have a long way to go in understanding and modifying the cardiovascular risks related to environmental factors such as air pollution and climate change, the cardiovascular community is starting to make headway. Understanding and integrating air pollution factors into risk assessment and management of cardiovascular disease will help patients and may even have larger unintended benefits. Health care systems should incorporate reducing their carbon footprint, in addition to health equity, cost efficiency, and care quality, to achieve a sustainable future.
